# Gait dynamics in mouse models of Parkinson's disease and Huntington's disease

**DOI:** 10.1186/1743-0003-2-20

**Published:** 2005-07-25

**Authors:** Ivo Amende, Ajit Kale, Scott McCue, Scott Glazier, James P Morgan, Thomas G Hampton

**Affiliations:** 1 Division of Medicine, Beth Israel Deaconess Medical Center, Harvard Medical School, Boston, MA 02215 USA; 2 The CuraVita Corporation, Boston, MA 02109 USA

**Keywords:** Gait variability, Gait, Mouse models, Neurodegeneration, Movement disorders, Amyotrophic Lateral Sclerosis, SOD1

## Abstract

**Background:**

Gait is impaired in patients with Parkinson's disease (PD) and Huntington's disease (HD), but gait dynamics in mouse models of PD and HD have not been described. Here we quantified temporal and spatial indices of gait dynamics in a mouse model of PD and a mouse model of HD.

**Methods:**

Gait indices were obtained in C57BL/6J mice treated with the dopaminergic neurotoxin 1-methyl-4-phenyl-1,2,3,6-tetrahydropyridine (MPTP, 30 mg/kg/day for 3 days) for PD, the mitochondrial toxin 3-nitropropionic acid (3NP, 75 mg/kg cumulative dose) for HD, or saline. We applied ventral plane videography to generate digital paw prints from which indices of gait and gait variability were determined. Mice walked on a transparent treadmill belt at a speed of 34 cm/s after treatments.

**Results:**

Stride length was significantly shorter in MPTP-treated mice (6.6 ± 0.1 cm vs. 7.1 ± 0.1 cm, *P *< 0.05) and stride frequency was significantly increased (5.4 ± 0.1 Hz vs. 5.0 ± 0.1 Hz, *P *< 0.05) after 3 administrations of MPTP, compared to saline-treated mice. The inability of some mice treated with 3NP to exhibit coordinated gait was due to hind limb failure while forelimb gait dynamics remained intact. Stride-to-stride variability was significantly increased in MPTP-treated and 3NP-treated mice compared to saline-treated mice. To determine if gait disturbances due to MPTP and 3NP, drugs affecting the basal ganglia, were comparable to gait disturbances associated with motor neuron diseases, we also studied gait dynamics in a mouse model of amyotrophic lateral sclerosis (ALS). Gait variability was not increased in the SOD1 G93A transgenic model of ALS compared to wild-type control mice.

**Conclusion:**

The distinct characteristics of gait and gait variability in the MPTP model of Parkinson's disease and the 3NP model of Huntington's disease may reflect impairment of specific neural pathways involved.

## Background

Disturbances in gait are symptomatic of Parkinson's disease (PD) and Huntington's disease (HD). Gait abnormalities in PD include shortened stride length [1,2], a dyscontrol of stride frequency [3], and postural instability [4]. Gait abnormalities in HD include reduced walking speed [5], widened stance width [6], reduced stride length [6,7], and sway [8]. Gait variability has also been shown to be significantly higher in patients with PD [9-11] and HD [7,9] compared to control subjects. Early detection of gait disturbances may result in earlier treatment. Therapies for PD and HD patients are often developed to ameliorate gait abnormalities [12,13]. Mouse models of PD and HD are used to understand the pathologies of the diseases and to accelerate the testing of new therapies to correct motor defects. Although spatial gait indices have been reported [14,15], gait dynamics in mouse models of PD and HD have not yet been described.

One common mouse model of PD is obtained by repeatedly administering the neurotoxin 1-methyl-4-phenyl-1,2,3,6-tetrahydropyridine (MPTP) [16-18]. MPTP causes damage of the nigrostriatal dopaminergic system [19], resulting in PD symptoms, including reduced stride length [14] and posture disturbances in mice [20]. One common mouse model of HD is obtained by repeatedly administering the mitochondrial toxin 3-nitropropionic acid (3NP) [21,22]. 3NP causes striatal neurodegeneration resulting in mild dystonia and bradykinesia comparable to HD in people [23,24].

Motor defects in MPTP-treated mice or 3NP-treated mice are often quantified using the rotarod test that measures the time a subject can balance on a rotating rod [25,26]. MPTP has been shown to reduce performance on the rotarod [27] or to have no effect on rotarod performance [17,28]. 3NP has been shown to reduce rotarod performance [29], or to have no effect on rotarod performance [30]. The swim test [31], balance beam test [32], and the pole test [33] have also been used to investigate the effects of MPTP and 3NP on motor function in mice. Results regarding motor dysfunction in the MPTP model of PD and the 3NP model of HD may vary due to the heterogeneity in protocols followed. Disparities in the degree of motor dysfunction have suggested that large doses of MPTP or 3NP may be required to detect motor defects after nigrostriatal damage [18,29,34].

Several studies in mouse models of PD and HD have described "gait" by estimating stride length [14], and stance width [15] determined by painting the animals' paws. Fernagut et al. reported that stride length is a reliable index of motor disorders due to basal ganglia dysfunction in mice [15]. Gait dynamics in humans, however, extend beyond the measure of stride length. Gait dynamics in humans include spatial indices such as stance width and foot placement angle. Gait dynamics in humans also include temporal indices, such as stride frequency, stride duration, swing duration, and stance duration.

Step-to-step gait variability in humans has also provided important information about possible mechanisms involved in neurodegenerative diseases, including PD and HD [7,9-11]. In patients with PD, higher step-to-step variability has been reported [9-11,35]. The stride length variability increased with the progression of PD suggesting that this index is useful in assessing the course of PD [10]. Hausdorff et al. demonstrated significantly higher variability in several gait indices, including stride duration and swing duration, in patients with PD and HD [9], and in subjects with amyotrophic lateral sclerosis (ALS) [36]. It has been proposed that a matrix of gait dynamic markers could be useful in characterizing different diseases of motor control [36]. Comparable analyses of gait and stride variability in mouse models of PD and HD have not yet been reported.

We recently described ventral plane videography using a high-speed digital camera to image the underside of mice walking on a transparent treadmill belt [37,38]. The technology generates "digital paw prints", providing spatial and temporal indices of gait. Here we applied ventral plane videography to study gait dynamics in the MPTP model of PD and the 3NP model of HD. We studied the C57BL/6 strain, which has been shown to be sensitive to both toxins [14,18,21,29]. Since PD, HD, and ALS share aspects of pathogenesis and pathology of motor dysfunction, we also studied gait dynamics in the SOD1 G93A transgenic mouse model of ALS [39] to compare gait variability in mouse models of basal ganglia disease to a mouse model of motor neuron disease.

## Methods

### Mice

Male C57BL/6J mice (7–8 weeks; ~22 gm) were purchased from The Jackson Laboratory (Bar Harbor, ME). Mice transgenic for the mutated human SOD1 G93A (TgN [SOD1-G93A]1Gur) (SOD1 G93A) and wild-type human SOD1 (TgN [SOD1]2Gur) wild-type controls) were purchased from The Jackson Laboratory (Bar Harbor, ME) when the mice were ~7.5 weeks old. Animals were maintained on a 12-hour light: 12-hour dark schedule with *ad libitum *access to food and water. Handling and care of mice were consistent with federal guidelines and approved institutional protocols.

### Experimental groups

#### MPTP

1-methyl-4-phenyl-1,2,3,6-tetrahydropyridine (MPTP) (Sigma-Aldrich, St. Louis, MO) dissolved in saline was administered 30 mg/kg i.p. to 7 mice every 24 hours for 3 days (MPTP-treated mice), based on previously published studies [40,41]. Equivolume (0.2 ml) of saline was administered i.p. to 7 control mice every 24 hours for 3 days (saline-treated mice).

#### 3NP

3-nitropropionic acid (3NP) (Sigma-Aldrich, St. Louis, MO) dissolved in saline was administered 3 times to 6 mice: 25 mg/kg i.p. twice, separated by 12 hours (cumulative dose of 50 mg/kg), then 25 mg/kg 24 hours later (cumulative dose of 75 mg/kg) (3NP-treated mice). Equivolume (0.2 ml) of saline was administered i.p. according to the same schedule to 6 control mice. The intoxication protocol was based on published studies [29,42], and our own pilot observations that higher doses resulted in high mortality rates or the inability of the mice to walk at all on the treadmill belt.

#### SOD1 G93A transgenic mice

To compare gait variability in the MPTP and 3NP mouse models of basal ganglia disease to a mouse model of motor neuron disease, we also examined gait in a mouse model of amyotrophic lateral sclerosis (ALS). Gait dynamics in SOD1 G93A mice were measured at ages ~8 weeks (n = 3), ~10 weeks (n = 3), ~12 weeks (n = 5), and ~13 weeks (n = 5), time points this model has been shown to exhibit motor dysfunction [43-45], and compared to wild-type control mice studied at ages ~8 weeks (n = 3), ~10 weeks (n = 3), ~12 weeks (n = 6), and ~13 weeks (n = 6).

### Gait dynamics

**Figure 1:**
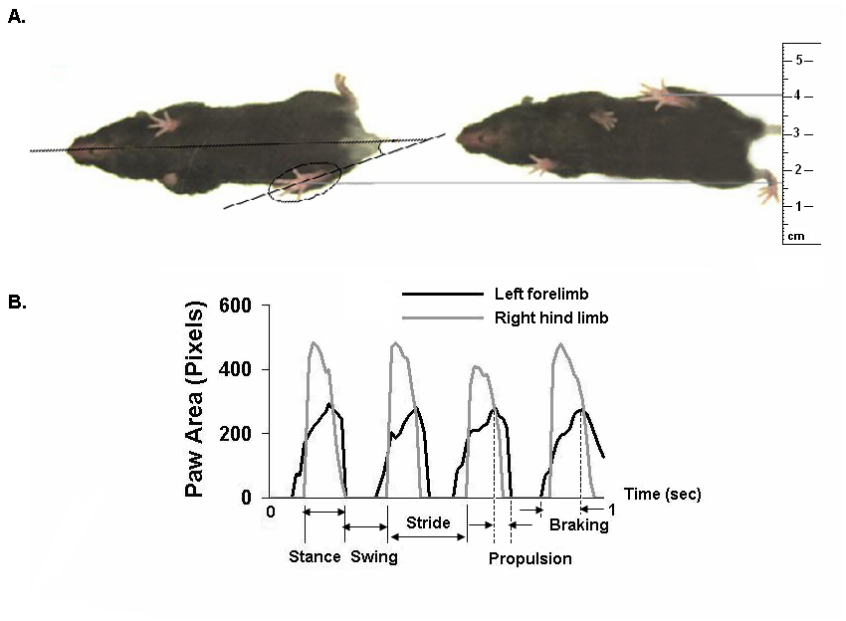
**Ventral view of walking saline-treated mouse**. **A. **Two images depicting the ventral view of a saline-treated C57BL/6J mouse on a transparent treadmill belt walking at a speed of 34 cm/s. The example on the left depicts full stance for the right hind limb, and the example on the right depicts sequential full stance for the left hind limb. Cartesian coordinates are used to determine stance width and paw placement angles for the forelimbs and hind limbs. **B. **Representative gait signals of the left forelimb and right hind limb of a saline-treated C57BL/6J mouse walking at a speed of 34 cm/s. Duration of stride, stance, and swing are indicated for the right hind limb. Duration of braking and propulsion are indicated for the left fore limb.

Gait dynamics were recorded using ventral plane videography, as previously described [37,38]. Briefly, we devised a motor-driven treadmill with a transparent treadmill belt. A high-speed digital video camera was mounted below the transparent treadmill belt. An acrylic compartment, ~5 cm wide by ~25 cm long, the length of which was adjustable, was mounted on top of the treadmill to maintain the mouse that was walking on the treadmill belt within the view of the camera. Digital video images of the underside of mice were collected at 80 frames per second. Each image represents 12.5 ms; the paw area indicates the temporal placement of the paw relative to the treadmill belt. The color images were converted to their binary matrix equivalents, and the areas (in pixels) of the approaching or retreating paws relative to the belt and camera were calculated throughout each stride. Plotting the area of each digital paw print (paw contact area) imaged sequentially in time provides a dynamic gait signal, representing the temporal record of paw placement relative to the treadmill belt (Figure 1). Each gait signal for each limb comprises a stride duration (stride time), which includes the stance duration when the paw of a limb is in contact with the walking surface, plus the swing duration when the paw of the same limb is not in contact with the walking surface. Stance duration was further subdivided into braking duration (increasing paw contact area over time) and propulsion duration (decreasing paw contact area over time) (Figure 1B).

Stride frequency was calculated by counting the number of gait signals over time. Stride length was calculated from the equation: *speed = stride frequency × stride length*. To obtain stance widths and paw placement angles at full stance, ellipses were fitted to the paws, and the centers, vertices, and major axes of the ellipses were determined. Forelimb and hind limb stance widths were calculated as the perpendicular distance between the major axes of the left and right paw images during peak stance. Gait data were collected and pooled from both the left and right forelimbs, and the left and right hind limbs.

Measures of stride-to-stride variability (gait variability) for stride length, stride time, and stance width were determined as the standard deviation and the coefficient of variation (CV). The standard deviation reflects the dispersion about the average value for a parameter. CV was calculated from the equation: *100 × standard deviation/mean value*.

Gait was recorded ~24 hours after each administration of saline or MPTP. Gait was recorded ~12 hours after the 1^st ^administration, and ~24 hours after the 2^nd ^and 3^rd ^administration of 3NP. Each mouse was allowed to explore the treadmill compartment for ~1 minute with the motor speed set to zero since our previous experience with C57BL/6J mice [37] indicated they do not require extended acclimatization to the treadmill. The motor speed was then set to 34 cm/s and images were collected. Approximately 3 seconds of videography were collected for each walking mouse to provide more than 7 sequential strides. Only video segments in which the mice walked with a regularity index of 100% [46] were used for image analyses. The treadmill belt was wiped clean between studies if necessary.

### Statistics

Data are presented as means ± SE. ANOVA was used to test for statistical differences among saline-treated, MPTP-treated, and 3NP-treated mice. When the F-score exceeded F_critical _for α = 0.05, we used *post hoc *unpaired Student's two-tailed *t*-tests to compare group means. Gait indices between forelimbs and hind limbs within the saline-treated mice were compared using Student's two-tailed *t*-test for paired observations. Gait indices between SOD1 G93A and wild-type control mice were compared using unpaired Student's two-tailed *t*-test. Differences were considered significant with *P *< 0.05.

## Results

### Gait in saline-treated mice

The ventral view of a C57BL/6J mouse walking on a transparent treadmill belt is shown in the upper panel of Figure 1 (and Additional file 1). Representative gait dynamics signals for the left forelimb and right hind limb of a saline-treated mouse walking at a speed of 34 cm/s are shown in the lower panel of Figure 1. Walking at a speed of 34 cm/s, C57BL/6J mice achieved ~5 steps every second, completed one stride within ~200 ms, and traversed ~7 cm with each step. The contributions of stance and swing durations to stride duration were ~55% (stance/stride) and ~45% (swing/stride) respectively. Forelimb stance width was significantly narrower than hind limb stance width (1.7 ± 0.1 cm vs. 2.4 ± 0.2 cm, *P *< 0.05). The paw placement angle of the hind limbs was significantly more open than the paw placement angle of the forelimbs (13.9 ± 1.6 vs. 2.6 ± 0.6, *P *< 0.05). Stride length variability of hind limbs was lower than of forelimbs (0.63 ± 0.08 cm vs. 0.78 ± 0.03 cm, *P *< 0.05). Likewise, stance width variability of hind limbs was lower than of forelimbs (0.14 ± 0.01 cm vs. 0.21 ± 0.02 cm, *P *< 0.05) in saline-treated mice walking on a treadmill belt at 34 cm/s.

### Gait in MPTP-treated mice

**Figure 2:**
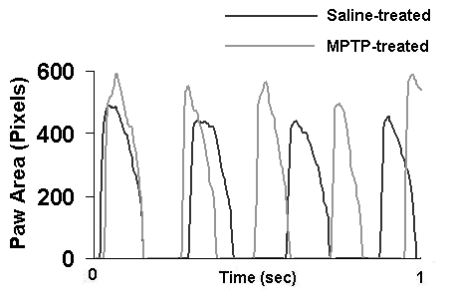
**Gait signals in a MPTP-treated mouse**. Gait signal of the right hind limb of a MPTP-treated mouse superimposed over the gait signal of the right hind limb of a saline-treated mouse. Stride frequency was higher in MPTP-treated mice compared to saline treated mice. Stance duration and swing duration were shorter in MPTP-treated mice compared to saline-treated mice.

Gait dynamics in MPTP-treated mice after 3 administrations of 30 mg/kg MPTP were significantly different than gait dynamics in saline-treated mice (Table 1 and Figure 2). Stride length was decreased in MPTP-treated mice compared to saline-treated mice (6.6 ± 0.1 cm vs. 7.1 ± 0.1 cm, *P *< 0.05) at a walking speed of 34 cm/s. Stride frequency was increased in MPTP-treated mice. Stride duration was significantly shorter in MPTP-treated mice (194 ± 1 ms vs. 207 ± 2 ms, *P *< 0.05). This was attributable to a shorter swing duration of the hind limbs (92 ± 3 vs. 104 ± 2 ms, *P *< 0.05), and a shorter stance duration of the forelimbs (116 ± 2 ms vs. 126 ± 2 ms, *P *< 0.05). The contributions of stance and swing to stride duration in MPTP-treated mice were not different than in saline-treated mice, despite the shorter stride duration. Forelimb stance width and hind limb stance width were comparable in MPTP-treated mice and saline-treated mice. The paw placement angles of the forelimbs and hind limbs of MPTP-treated mice were not different than in saline-treated mice. Figure 2 illustrates the gait signal from the right hind limb of a MPTP-treated mouse superimposed over the gait signal from the right hind limb of a saline-treated mouse.

**Table 1:** Gait dynamics in saline-treated, MPTP-treated (90 mg/kg cumulative dose), and 3NP-treated (75 mg/kg cumulative dose) mice walking on a treadmill belt at a speed of 34 cm/s.

	Saline (n = 7)	MPTP (n = 7)	3NP (n = 3)
Stride Length (cm)	7.1 ± 0.1	6.6 ± 0.1*	7.3 ± 0.1
Stride Frequency (Hz)	5.0 ± 0.1	5.4 ± 0.1*	4.9 ± 0.1
Stride Duration (ms)	207 ± 2	194 ± 1*	217 ± 5
% Stance Duration	54.3 ± 0.9	55.9 ± 1.1	59.4 ± 2.3*
% Swing Duration	45.7 ± 0.9	44.1 ± 1.1	40.6 ± 2.3*
Forelimb Stance Width (cm)	1.7 ± 0.1	1.6 ± 0.1	1.7 ± 0.1
Forelimb Paw Placement Angle (°)	2.6 ± 0.6	2.6 ± 0.4	3.5 ± 1.1
Hind limb Stance Width (cm)	2.4 ± 0.2	2.2 ± 0.1	2.8 ± 0.2
Hind limb Paw Placement Angle (°)	13.9 ± 1.6	10.8 ± 1.3	15.2 ± 1.0

Means ± SE. *P < 0.05, compared to saline-treated mice.

**Figure 3:**
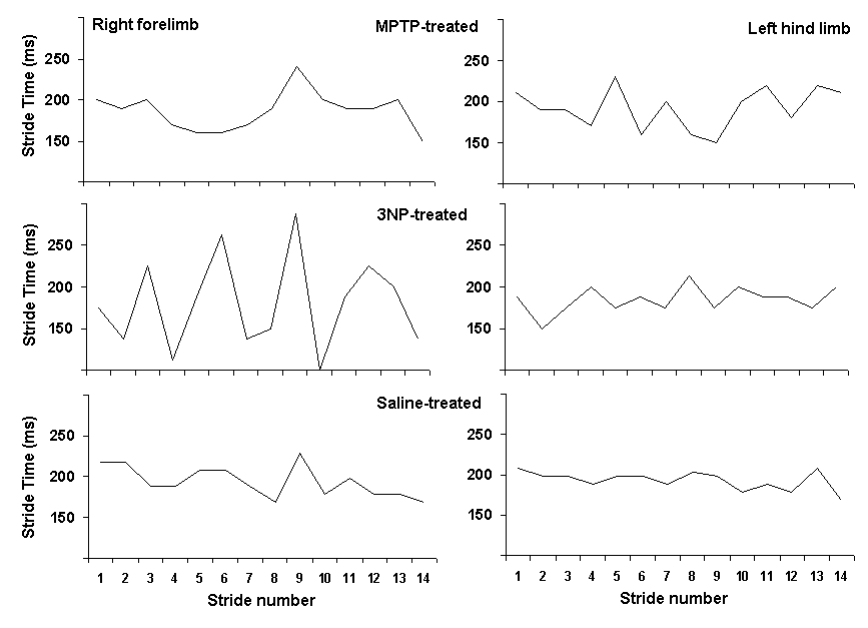
**Stride time dynamics**. Examples of stride time (gait cycle duration) in MPTP-treated, 3NP-treated, and saline-treated mice of forelimbs (left panels) and hind limbs (right panels). In saline-treated animals, forelimb stride variability was higher than hind limb stride variability. MPTP-treated and 3NP-treated mice exhibited significantly higher stride variability. The coefficient of variation (CV), a measure of stride-to-stride variability, was highest in the forelimbs of 3NP-treated mice.

Stride time dynamics for 14 sequential strides in a MPTP-treated mouse are shown in the top panel of Figure 3. For comparison, stride time dynamics in a 3NP-treated mouse are illustrated in the middle panel, and in saline-treated mouse in the bottom panel of Figure 3. Gait variability was significantly higher in MPTP-treated mice after 3 treatments compared to saline-treated mice. Stride length variability of the forelimbs was higher in MPTP-treated than in saline-treated mice (0.91 ± 0.04 cm vs. 0.78 ± 0.03 cm, *P *< 0.05). Stride length variability of the hind limbs, however, was not different in MPTP-treated mice. The coefficient of variation (CV) of forelimb stride length was significantly higher in MPTP-treated than in saline-treated mice (13.6 ± 0.8 % vs. 11.1 ± 0.8 %, *P *< 0.05). The CV of hind limb stride length was somewhat higher in MPTP-treated than in saline-treated mice (10.0 ± 1.5 % vs. 8.0 ± 0.7 %, NS).

Stance width variability of the forelimbs was significantly higher in MPTP-treated than in saline-treated mice (0.26 ± 0.01 cm vs. 0.21 ± 0.02 cm, *P *< 0.05). Stance width variability of the hind limbs was higher in MPTP-treated than in saline-treated mice (0.20 ± 0.02 cm vs. 0.14 ± 0.01 cm, *P *< 0.05). The CV of forelimb stance width was higher in MPTP-treated than in saline-treated mice (16.7 ± 1.3 % vs. 12.3 ± 1.2 %, *P *< 0.05). The CV of hind limb stance width was higher in MPTP-treated than in saline-treated mice (9.1 ± 1.1 % vs. 5.9 ± 0.5 %, *P *< 0.05).

### Gait in 3NP-treated mice

Stride length, stride frequency, stance duration, and swing duration were not affected by 3NP after the 1^st ^and 2^nd ^administrations of 25 mg/kg. The paw placement angle of the hind limbs, however, was significantly more open in 3NP-treated mice (n = 6) compared to saline-treated mice (16.6 ± 1.2° vs. 12.4 ± 1.5°, *P *< 0.05) after the 2^nd ^administration of 3NP (cumulative dose of 50 mg/kg). Stance width variability of the forelimbs, moreover, was higher in 3NP-treated than in saline-treated mice (0.28 ± 0.01 cm vs. 0.22 ± 0.02 cm, *P *< 0.05) after the 2^nd ^administration of 3NP. The CV of forelimb stance width was higher in 3NP-treated than in saline-treated mice (15.0 ± 1.2 % vs. 11.7 ± 0.6 %, *P *< 0.05) after the 2^nd ^administration of 3NP. Neither stride length variability nor stance width variability of the hind limbs was affected after the 2^nd ^administration of 3NP (cumulative dose of 50 mg/kg).

After the 3^rd ^administration of 3NP (cumulative dose of 75 mg/kg), half of the 3NP-treated mice could not walk on the treadmill belt at a speed of 34 cm/s. Forelimb gait indices in the three 3NP-treated mice that could walk on the treadmill belt were similar to saline-treated mice. Hind limb gait indices, however, were affected in the three 3NP-treated mice that could walk on the treadmill belt. The hind limb stance width (2.8 ± 0.2 cm) and paw placement angle (15.2 ± 1.0°) in the 3NP-treated mice that could walk on the treadmill belt (n = 3) tended to be greater than in saline-treated mice. The percentage of stride spent in stance was significantly greater in 3NP-treated mice than in saline-treated mice (59.4 ± 2.3% vs. 54.3 ± 0.9 %, *P *< 0.05). The percentage of stance duration spent in propulsion (propulsion/stance) was greater of the hind limbs in 3NP-treated mice than in saline-treated mice (45.2 ± 2.5 % vs. 40.2 ± 0.9 %, *P *< 0.05). This was at the expense of a smaller contribution of swing to stride duration (40.6 ± 2.3 % vs. 45.7 ± 0.9 %, *P *< 0.05).

Stride length variability of the forelimbs, moreover, was significantly higher in the three 3NP-treated mice that could walk than in saline-treated mice (1.31 ± 0.09 cm vs. 0.87 ± 0.07 cm, *P *< 0.05). Stance width variability of the forelimbs was also higher in 3NP-treated than in saline-treated mice (0.31 ± 0.04 cm vs. 0.22 ± 0.01 cm, *P *< 0.05). The CV of forelimb stride length was higher in 3NP-treated than in saline-treated mice (17.9 ± 1.6 % vs. 11.8 ± 0.8 %, *P *< 0.05) (Figure 3). The CV of forelimb stance width was higher in 3NP-treated than in saline-treated mice (17.3 ± 2.4 % vs. 11.7 ± 0.6 %, *P *< 0.05). Hind limb stride length variability and hind limb stance width variability were not different in the 3NP-treated mice that could walk on the treadmill belt compared to saline-treated mice.

### Hind limb gait failure in 3NP-treated mice

**Figure 4:**
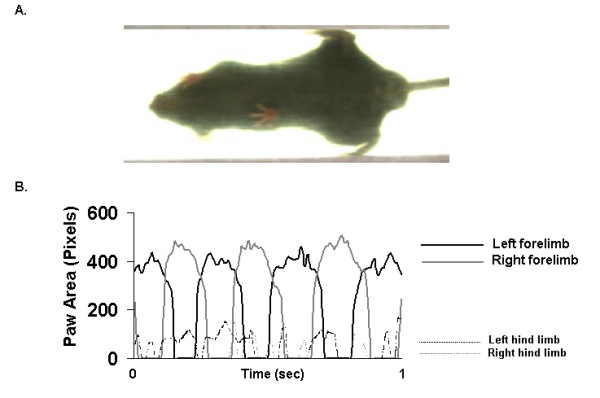
**Ventral view of a 3NP-treated mouse attempting to walk**. **A. **The ventral view of a 3NP-treated mouse attempting to walk on the treadmill belt moving at a speed of 34 cm/s but failing to engage the hind limbs in coordinated stepping. This animal braced its hind paws onto the base of the sidewalls of the walking compartment avoiding the moving treadmill belt. Only the forelimbs execute coordinated stepping sequences. **B. **Gait signals of the left and right forelimbs of a 3NP-treated mouse demonstrating coordinated stepping, despite hind limb failure of stepping. The signals of left and right hind limbs are not coordinated and reflect artefacts associated with the belt contacting the braced paws.

Two 3NP-treated mice that could not walk on the moving treadmill belt at a speed of 34 cm/s, however, attempted to walk, but failed to engage the hind limbs in coordinated stepping. Rather, these mice braced their hind paws onto the base of the sidewalls of the walking compartment (Figure 4, upper panel; Additional file 2), avoiding the moving treadmill belt. The forelimbs of these 3NP-treated mice, however, executed coordinated stepping on the moving treadmill belt. Forelimb stride dynamics in these 3NP-treated mice did not differ significantly from saline-treated mice and the three 3NP-treated mice that were able to walk on the treadmill belt at 34 cm/s (Figure 4, lower panel). Despite the limitation of these 3NP-treated mice to only execute forelimb stepping, stride length of forelimbs was 7.1 ± 0.1 cm, stride frequency was 5.0 ± 0.1 Hz, and stance duration was 133 ± 5 ms, all values similar to forelimb gait indices in saline-treated mice.

### Gait in SOD1 G93A transgenic mice

Stride length was significantly greater in SOD1 G93A mice (n = 5) than in wild-type mice (n = 6) at ~12 weeks and ~13 weeks of age. At ~12 weeks of age, stride length was significantly increased in SOD1 G93A mice compared to wild-type control mice (7.1 ± 0.1 cm vs. 6.7 ± 0.1 cm, *P *< 0.05). Stride frequency was lower in SOD1 G93A mice (5.0 ± 0.1 vs. 5.4 ± 0.1 Hz, *P *< 0.05), and stride duration was longer compared to wild-type control mice (210 ± 2 vs. 197 ± 3 ms, *P *< 0.05) at ~12 weeks of age. At ~13 weeks of age, stride length remained significantly increased in SOD1 G93A mice compared to wild-type control mice (7.1 ± 0.1 cm vs. 6.8 ± 0.1 cm, *P *< 0.05). Stride frequency remained lower in SOD1 G93A mice (5.0 ± 0.1 vs. 5.3 ± 0.1 Hz, *P *< 0.05), and stride duration remained longer compared to wild-type control mice (209 ± 2 vs. 198 ± 3 ms, *P *< 0.05) at ~13 weeks of age.

Gait variability was monitored in SOD1 G93A mice at ~8 weeks, ~10 weeks, ~12 weeks, and ~13 weeks of age, coinciding with the appearance of motor dysfunction reported in this model [43-45]. Gait variability was not different in SOD1 G93A mice compared to wild-type control mice at age ~8 weeks, ~10 weeks, ~12 weeks, and ~13 weeks. Stride length variability of the forelimbs and hind limbs were comparable between SOD1 G93A mice and wild-type control mice at all ages studied. Stance width variability of the forelimbs and hind limbs were also comparable between SOD1 G93A and wild-type control mice at age ~8 weeks, ~10 weeks, ~12 weeks, and ~13 weeks.

## Discussion

Gait disturbances are characteristic of Parkinson's disease, Huntington's disease, and amyotrophic lateral sclerosis. Gait reflects several variables, including balance, proprioception, and coordination. There are several mouse models of PD [20,47] and HD [22,48-50], and one widely studied model of ALS [39,43-45]. Mouse models that replicate PD, HD, and ALS symptoms could improve understanding of their pathogenesis and treatment. Gait variability indices are increasingly being recognized as important markers of neurological diseases [4,9-11,36]. We found gait disturbances, including increased gait variability, in the MPTP-treated mouse model of PD and the 3NP-treated mouse model of HD, which may be the consequence of the affected neural pathways. Gait variability was not increased, however, in the SOD1 G93A transgenic mouse model of ALS.

### Gait in MPTP-treated mice

The MPTP-treated mouse model of PD has been extensively studied for its ability to injure the nigrostriatal dopaminergic system, damage neurons, and deplete the brain of dopamine [16-18]. Several studies have described motor function disturbances in MPTP-treated mice to relate the deficits to symptoms in humans with PD. Motor function tests in MPTP-treated mice have included grip strength [40], the ability of the animals to balance on a rotating rod [27,40], and swimming performance [51]. MPTP significantly affects locomotor activity [17,40,52] and motor performance [17,20,28,51], thus providing functional readouts to test potential therapies. Shortened stride length is one of the cardinal features of PD [1,4,11], yet reports of reduced stride length in MPTP-treated animals are sparse. Fernagut et al., using the paw-inking method, measured stride length in mice one week after acute MPTP intoxication [14] and concluded that stride length was a reliable indicator of basal ganglia dysfunction. Smaller doses of MPTP (3 mg/kg) were also found to significantly reduce stride length in rats [53]. The difficulties associated with the paw-inking method and the variability in overground walking speeds in mice [54] have possibly limited reports of stride length in MPTP-treated mice. Using digital paw prints obtained by ventral plane videography, we found that stride length was significantly decreased in MPTP-treated mice after 3 days of administration (i.p. 30 mg/kg/day).

Gait indices, including stride duration, stance duration, swing duration, and stride length, change with changes in walking speed. We eliminated the confounding effects of differences in walking speed on gait dynamics by setting the motorized treadmill belt to 34 cm/s for all mice. Accordingly, since stride length was decreased in MPTP-treated mice, stride frequency was increased and stride duration was decreased in forelimbs and hind limbs of MPTP-treated mice. A decrease in stride duration can be attained by decreases in stance duration and swing duration. We found that the decrease in stride duration in MPTP-treated mice was attained by significantly shorter hind limb swing duration and forelimb stance duration. A reduction of the stance duration may result in a shorter time for limb muscles to be activated for stabilization [55]. This may account for the significant increase in stride-to-stride variability observed in MPTP-treated mice. Fleming et al. studied mice overexpressing wild-type human α-synuclein (ASO mice), a model of early onset familial PD [47]. The authors found that although stride length was comparable to control mice, stride frequency and stride length variability were increased in ASO mice [47]. ASO mice did not exhibit a loss of dopaminergic neurons, but developed accumulation of α-synuclein in the nigrostriatal system and show enhanced sensitivity of nigrostriatal neurons to MPTP administration [47].

### Gait in 3NP-treated mice

Gait dynamics in 3NP-treated mice were difficult to study. Aggressive doses of 3NP resulted in high mortality or the inability of the mice to walk at all on the treadmill belt (data not shown). The earliest effect of 3NP (12 hours after 1^st ^dose of 25 mg/kg) on gait was an increase in forelimb stride length variability. Subsequent gait disturbances included increased gait variability of the forelimbs and eventual failure of hind limb stepping. Our findings of different effects of 3NP on gait dynamics of forelimbs and hind limbs are in accordance with previous motor behavioral assessments in 3NP-treated animals [29,56]. Fernagut et al. found no differences in stride length of forelimbs and hind limbs after a cumulative dose of 3NP (340 mg/kg) [29]. With a cumulative dose of 560 mg/kg of 3NP, forelimb stride length was comparable to saline-treated mice, but hind limb stride length was shortened [29]. Administration of 3NP may affect hind limb gait dynamics differently than forelimb gait dynamics via different effects on the neostriatum and the nucleus accumbens [14,57]. Shimano et al. showed that hind limb muscles in 3NP-treated rats became hypotonic with low voltage electromyogram activity and impaired movement [58]. Activation of the motor program required for the two 3NP-treated mice that braced their hind limbs against the inside walls of the walking compartment while simultaneously maintaining coordinated gait of the forelimbs [59] may suggest that 3NP-induced cognitive defects [60] did not contribute to the gait disturbances in 3NP-treated animals.

Lin et al. reported that stride length and stance width in a knock-in mouse model of HD did not differ from wild-type mice [48]. Stride length variability and stance width variability were higher, however, in the mutants [48]. In a transgenic mouse model for HD, R6/2 mice exhibited unevenly spaced shorter strides, staggering movements, and an abnormal step sequence pattern [49]. No significant abnormalities in stride length were observed in the R6/1 HD transgenic mouse [50]. The significantly higher gait variability of the forelimbs we observed in 3NP-treated mice may reflect the jerky and highly variable arm movements in HD gene carriers and patients with HD [61]. Taken together, increases in forelimb stride variability appear to be more characteristic of motor control deficits in early HD than decreases in stride length.

### Gait in SOD1 G93A mice

Impaired performance in SOD1 G93A mice in some motor function tests have been observed at ~8 weeks of age [45]. Others have reported motor impairments in SOD1 G93A mice at ~11–16 weeks of age [43,44]. It was of interest, therefore, to find that stride length was significantly longer in SOD1 G93A mice compared to wild-type mice at ~12 weeks and ~13 weeks of age. Increased stride length is often associated with increased amplitude of electromyogram activity and enhanced motor performance. Gurney et al. first described significantly shorter stride length in SOD1 G93A mice with severe pathological changes in the late stage of disease [39]. Puttaparthi et al. also reported significantly shorter stride length in SOD1 G93A mice at ~24 weeks of age [44]. The reported decrease in stride length at later stages could be due to muscle weakness, fatigue, and motor neuron loss. The data of Puttaparthi et al. also indicate, however, that stride length in SOD1 G93A mice may tend to be longer at ~16 weeks of age [44]. Wooley et al., moreover, recently reported significantly longer stride duration in SOD1 transgenic mice compared to wild-type mice walking on a treadmill at 23 cm/s at 8 and 10 weeks of age [62], which would mean that SOD1 transgenic mice had significantly longer stride lengths at 8 and 10 weeks of age. It is notable that patients with ALS who walked overground at speeds comparable to healthy subjects also had longer stride duration [36]. One explanation for the increased stride length in the presymptomatic SOD1 G93A mice we observed walking 34 cm/s could be aberrant electrical activity of the muscles involved in treadmill walking. Kuo et al., in fact, identified significantly elevated intrinsic electrical excitability in cultured embryonic and neonatal mutant SOD1 G93A spinal motor neurons [63]. Dengler et al. surmised that new motor unit sprouting and resulting increases of twitch force could compensate for the loss of motor neurons in patients with early stages of ALS [64]. To our knowledge, there are no reports regarding stride length in patients with ALS walking on a treadmill. An early indication of ALS could be an increase in stride length.

### Gait variability indices

The CVs of stride length and stance width in healthy humans are ~3–6% and ~14–17%, respectively [65,66]. The CV of stride time in humans with intact neural control is <3%, and is significantly higher in patients with PD, HD, and ALS [36]. Stride time variability was highest in patients with HD [36]. The CV for stride length in saline-treated C57BL/6 mice is higher than in healthy humans, but the CV for stance width is comparable. Stride length may be determined predominantly by gait-patterning mechanisms, whereas stance width may be determined by balance-control mechanisms [67]. Stride length may be more variable in mice because of a greater number of gait patterns [37]. Gait variability may also be high in mice walking on a treadmill belt at a speed of 34 cm/s compared to mice walking overground at preferred speeds.

We found that gait variability of the forelimbs in mice was significantly higher than gait variability of the hind limbs. This may be attributable to the role of the forelimbs in balance and navigation [68,69]. We further found that the MPTP mouse model recapitulated the higher gait variability in patients with PD, as evidenced by a significant increase in stride length variability of the forelimbs and a significant increase in stance width variability of the forelimbs and hind limbs. We also found that the 3NP mouse model may reflect the higher gait variability in patients with HD, as evidenced by a significant increase in forelimb stride length variability and stance width variability. We found that gait variability of the forelimbs was highest in 3NP-treated mice, in parallel with the higher gait variability in patients with HD as compared to patients with PD [35]. The higher forelimb stride length variability in 3NP-treated mice may reflect the jerky movements of arms in HD patients [61]. Although pathology of PD and HD involve different portions of the basal ganglia, postural instability is common to both diseases. Postural instability was characteristic of MPTP-treated and 3NP-treated mice. Increased stride length and step width variability of the hind limbs was more characteristic in the MPTP model of PD than in the 3NP-model of HD. The more open paw placement angle of the hind limbs in 3NP-treated mice was not accompanied by higher stance width variability and stride length variability. Moreover, the eventual failure of the hind limbs in 3NP-treated mice (75 mg/kg cumulative dose) to engage in coordinated stepping was not preceded by changes in hind limb gait variability (50 mg/kg cumulative dose). We did not find an increase in gait variability in transgenic SOD1 G93A mice. Neither forelimb nor hind limb stride length variability or stance width variability in SOD1 G93A mice were different than in wild-type controls at ~12 weeks or ~13 weeks, ages when motor function deficiencies have been observed. In patients, gait variability was shown to be higher with well-established ALS [36]. We do not yet know if gait variability increases in SOD1 G93A mice as the disease progresses. Our findings suggest, however, that gait variability is not increased in the early stages of motor neuron disease. Differences in gait variability among MPTP-treated, 3NP-treated, and SOD1 G93A mice may reflect differences in neuropathology.

## Limitations

We do not know the long-term effects of extended administrations of MPTP or 3NP on gait dynamics. Different schedules of neurotoxin administration result in differences in the mechanisms of neuronal death [34,70], which could affect gait. We did not observe morbidity and mortality in the MPTP-treated mice. Results in 3NP-treated mice, however, were variable, consistent with reports of significant inter-animal variation in response to 3NP toxicity [71]. MPTP- and 3NP-induced neuronal damage in mice are age-dependent [72,73], and both toxins have systemic effects, including the heart [42,74]. Since no postmortem analyses were performed demonstrating neurodegeneration, the pathogenesis of the gait disturbances is unclear. We did not measure striatal dopamine; previous reports indicate, however, that 30 mg/kg/day MPTP for 3 days reduce striatal dopamine by >50% [18,20]. Neither the MPTP nor the 3NP toxin models exactly replicate the pathological phenomena of PD and HD. Future studies could compare gait dynamics in different chemically-induced models and genetic models of PD and HD. We did not consider effects of habituation to treadmill walking [61] on gait indices. Gait dynamics are strain-dependent [75], making it difficult to compare gait dynamics in the SOD1 G93A transgenic mouse model of ALS, which is a mix of C57BL/6 and SJL mice, to gait in the MPTP-treated and 3NP-treated C57BL/6 mice.

## Conclusion

MPTP-treated mice demonstrated significant gait disturbances, including shortened stride length, increased stride frequency, and increased stride-to-stride variability, symptoms characteristic of patients with Parkinson's disease. 3NP-treated mice demonstrated an increased forelimb stride-to-stride variability and a more open paw placement angle of the hind limbs. Gait failure in 3NP-treated mice resulted from an inability of the hind limbs to engage in stepping while forelimb gait remained intact. Gait variability was not significantly higher in SOD1 G93A mice, a model of motor neuron disease, compared to wild-type control mice. The present study provides a basis for additional studies of gait and gait variability in mouse models of PD, HD, and ALS.

## Competing interests

Thomas G. Hampton is owner of Mouse Specifics, Inc., a company organized to commercialize the gait imaging technology described in the methods.

## Authors' contributions

IA participated in data collection, analyses, interpretation, and manuscript preparation.

AK assisted in the design and development of the gait imaging system and developed the software for analyses of gait data via ventral plane videography. AK also participated in the collection and analyses of data. SM participated in the design of the walking compartment for mice on the moving treadmill belt, and participated in the collection of data and in manuscript preparation. SG participated in the design of the treadmill system, automation of image acquisition and modulation of treadmill belt speed. SG also participated in manuscript preparation. JPM participated in study design, pharmacology and physiology, data interpretation, and manuscript review. TGH designed the study, and participated in the collection and analyses of data, data interpretation, and manuscript preparation and submission.

## Supplementary Material

Additional File 1 Movie of the ventral view of a C57BL/6J saline-treated mouse walking at a speed of 34 cm/s. File is playable using Windows Media Player. Click here for file

Additional File 2 Movie of the ventral view of a 3NP-treated (cumulative dose 75 mg/kg) C57BL/6J mouse attempting to walk at a speed of 34 cm/s, demonstrating coordinated gait of the forelimbs but gait failure of the hind limbs. Compare this to the coordinated gait of the forelimbs and hind limbs in a saline-treated C57BL/6J mouse (Additional file 1). Files areplayable using Windows Media Player. Click here for file
